# Venetoclax combined with azacitidine in blastic plasmacytoid dendritic cell neoplasm: a case report and comprehensive review on the current and future treatment

**DOI:** 10.3389/fmed.2024.1425833

**Published:** 2024-07-17

**Authors:** Xiaoning Wang, Jiashuo Guo, Yan Liu, Na Zheng, Shaohan Xu, Lianhui Wu, Ruirui Yuan, Liying Xue, Jie Li

**Affiliations:** ^1^Department of Hematology, Hebei General Hospital, Shijiazhuang, China; ^2^Laboratory of Pathology, Hebei Medical University, Shijiazhuang, China

**Keywords:** blastic plasmacytoid dendritic cell neoplasm, BCL2 inhibitor, venetoclax, hypomethylating agent, azacitidine, combination therapy

## Abstract

Blastic plasmacytoid dendritic cell neoplasm (BPDCN) is an extremely rare hematological malignancy with a highly aggressive behavior and median survival of <2 years. Especially, most BPDCN patients present with extensive and non-specific skin lesions, usually leading to misdiagnosis as a skin disease and delay therapy. As for treatment, most patients with BPDCN experience relapse shortly after treatment with the traditional regimens. The alleviation of skin symptoms reflects the effects of clinical treatments. Herein, we report a case of a 71-year-old man with intermittent and gradually expanding skin lesions over his chest, abdomen, and back for 1 year. On admission, physical examination revealed extensive skin lesions and multiple enlarged lymph nodes. Laboratory examinations showed pancytopenia and numerous malignant cells in the peripheral blood smear (60%), bone marrow aspirate smear (73.5%). Immunophenotyping using flow cytometry and immunohistochemistry presented large numbers of BPDCN cells in the bone marrow, cervical lymph nodes and dermal tissue. PET/CT revealed multiple enlarged lymph nodes and splenomegaly. Once the diagnosis was identified as BPDCN, the patient began treatment with the oral BCL2 inhibitor venetoclax and subcutaneously administered azacitidine. After the first course, skin lesions reduced markedly and complete remission was achieved in the bone marrow. Our study and current cumulative data according to reviewing systematically suggest that venetoclax combined with azacitidine is safe, effective, and applicable in the treatment of BPDCN, especially for elderly relapsed/refractory patients. This study, therefore, significantly contributes to the literature on the current and future treatment for BPDCN.

## 1 Introduction

Blastic plasmacytoid dendritic cell neoplasm (BPDCN) originates from the malignant clonal expansion of precursor plasmacytoid dendritic cells (pDCs) and is an extremely rare hematological malignancy with a highly aggressive behavior and poor prognosis with a median survival of <2 years (1, 2). The incidence rate of BPDCN in the American population was only 0.09/100,000 in elderly people aged >60 years, whereas it was 0.02/100,000 in young adult aged 20–59 years (1). Its heterogeneous clinical manifestations include involvement of the skin, bone marrow (BM), blood, lymph nodes (LN), and central nervous system (CNS)/cerebrospinal fluid (CSF). Surprisingly, approximately 85% of the patients with BPDCN present with non-specific skin lesions (3–5). Due to the heterogeneous manifestations, misdiagnosis often occurs and leads to delay in the treatment. Even though the patients were treated with conventional regimens (widely used for other hematological malignancies), most cases relapse and develop drug resistance within a short time (6–8). In 2018, a novel targeted CD123 antibody (tagraxofusp) was approved as the first-line treatment for patients with BPDCN; however, serious adverse events, such as liver injury and capillary leak syndrome, limit its clinical application (9). It is widely known that hematopoietic stem cell transplantation (HSCT) can improve the survival outcomes of patients. Despite this, most elderly patients cannot undergo HSCT owing to their poor physical condition. To begin to formally address the state of the field, the North American BPDCN Consortium (NABC) was formed and gave a consensus treatment approach for the first time this year (10). However, a greater understanding of different regimens used in BPDCN is very important and remains an important goal for the worldwide consortium.

BCL2 (antiapoptotic protein B-cell leukemia/lymphoma-2) inhibitors (venetoclax) and hypomethylating agents (HMAs), such as azacitidine, are used to treat hematological malignancies (10–13). Previous studies have demonstrated that venetoclax combined with azacitidine can increase the rate of complete remission (CR) and prolong the survival of patients with acute myeloid leukemia (AML) (14). A recent study also reported that BCL2 is highly expressed in BPDCN cells (15), and its inhibitor is usually administered as a single regimen or in combination with a hyper-CVAD regimen/HMA. Importantly, when treated with a BCL2 inhibitor and HMA, patients with BPDCN showed a rapid clinical response and few side effects. Thus, this combination appears to have promising therapeutic potential for the treatment of BPDCN. Herein, we describe a case of BPDCN in a 71-year-old man with intermittent and gradually expanding skin lesions. After the combination treatment of venetoclax and azacitidine, the skin lesions gradually reduced in size and disappeared markedly. Besides that, the other main content is to comprehensively review the previous literatures before January 2023 and further give the analysis and summary on these studies. This systematically review will make a significant contribution to the current and future treatment on BPDCN.

## 2 Case description

A 71-year-old man was admitted to Hebei General Hospital (Shijiazhuang) on 5 May 2022 because of intermittent skin lesions on his chest, abdomen, and back for 1 year and lymphadenopathy for 7 months. One year ago, the patient was diagnosed as allergic rash in a county hospital because of the skin lesions. After treated with diphenhydramine, dexamethasone and ebastine, the skin lesions faded slowly. However, the skin lesions reappeared and were accompanied by left abdominal pain 7 months prior to the presentation. Examination at the county hospital revealed pancytopenia and many enlarged lymph nodes in the enterocoelia and retroperitoneum. After symptomatic and supportive therapy, the pain was relieved, and the skin lesions dissipated again. Two weeks prior, the patient was admitted to our hospital with skin lesions, extreme fatigue, bilateral neck and inguinal lymph node enlargement, and an increased number of enterocoelia lymph nodes. His past medical history was unremarkable.

**Figure 1:**
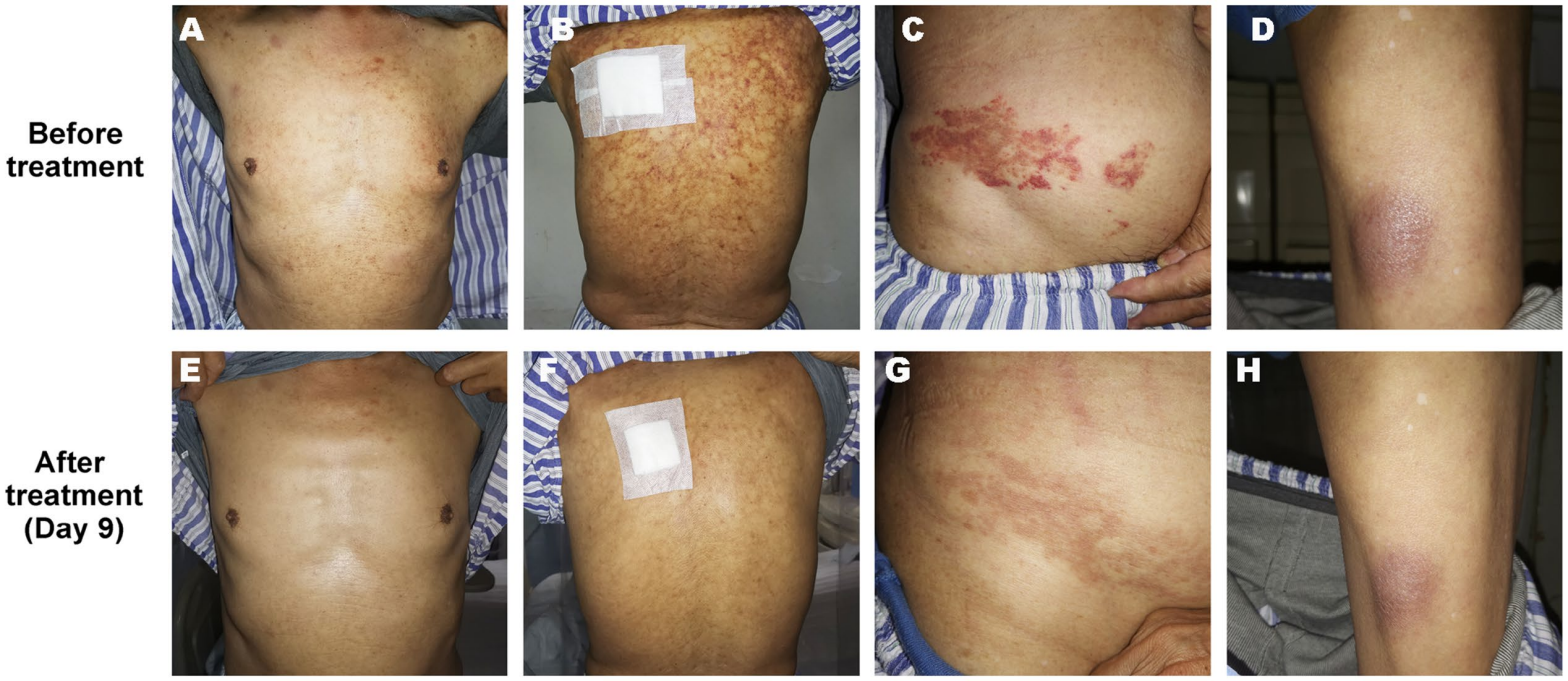
Skin lesion on the chest **(A,E)**, back **(B,F)**, abdomen **(C,G)**, and right inner thigh **(D,H)** of the patient before and after treatment (Day 9).

**Figure 2:**
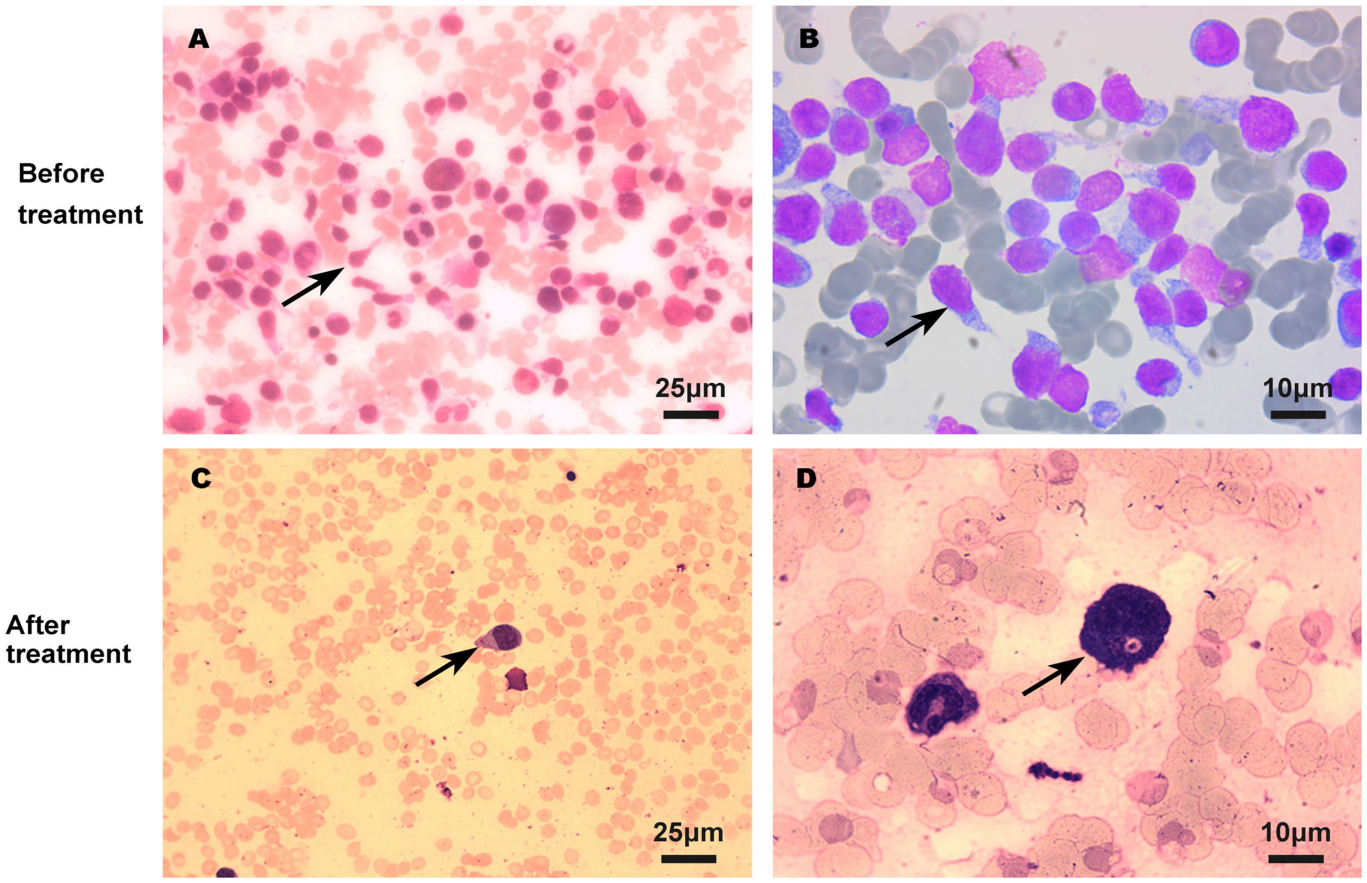
BPDCN cells in bone marrow smear before **(A,B)** and after **(C,D)** treatment using Wright’s staining. The quantity of BPDCN cells significantly decreased in bone marrow smear after treatment.

**Figure 3:**
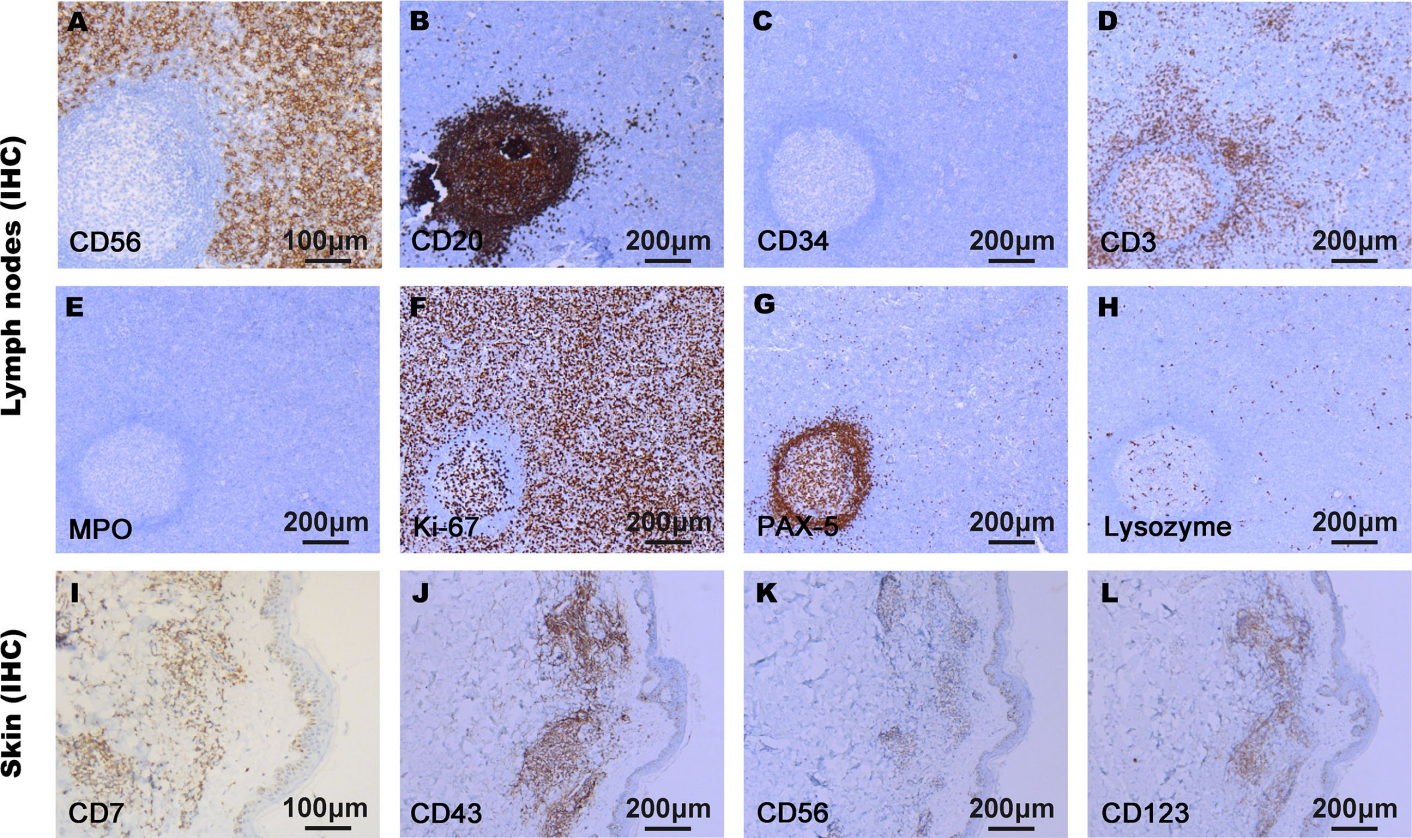
Immunophenotypes of malignant cells in lymph nodes **(A–H)** and skin tissue **(I–L)** using immunohistochemistry (IHC). The tumor cells proliferated actively. CD7, CD43, CD56, CD123, and Ki-67 were positively expressed in BPDCN cells, and CD3, CD20, CD34, MPO, Lysozyme, and PAX-5 were negatively expressed in BPDCN cells.

**Figure 4:**
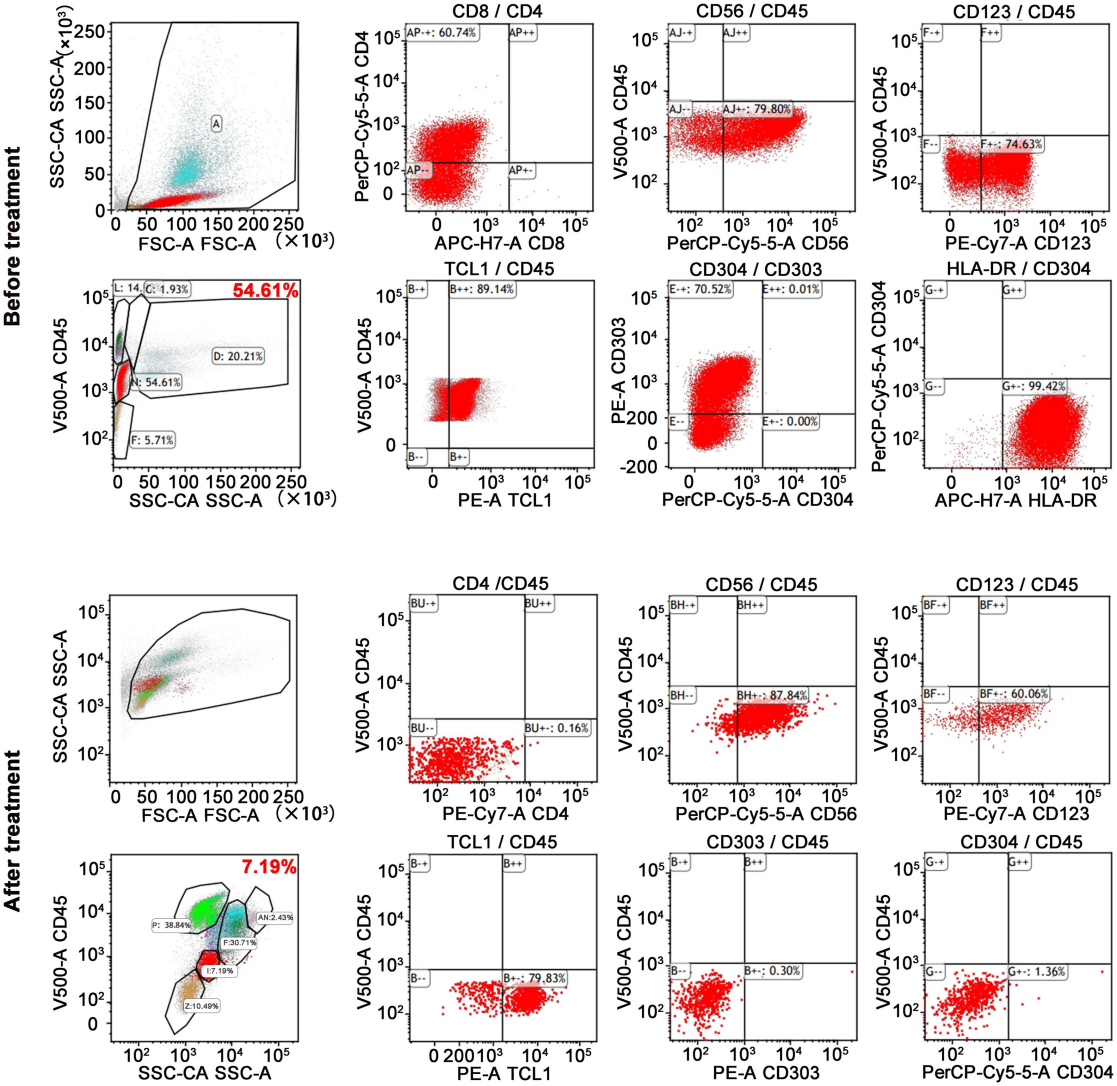
FCM results showed a lot of BPDCN cells in bone marrow. BPDCN cells were positive for CD4, CD56, CD123, TCL1, and CD303. BPDCN cells significantly decreased in bone marrow after treatment (from 54.61 to 7.19%).

**Figure 5:**
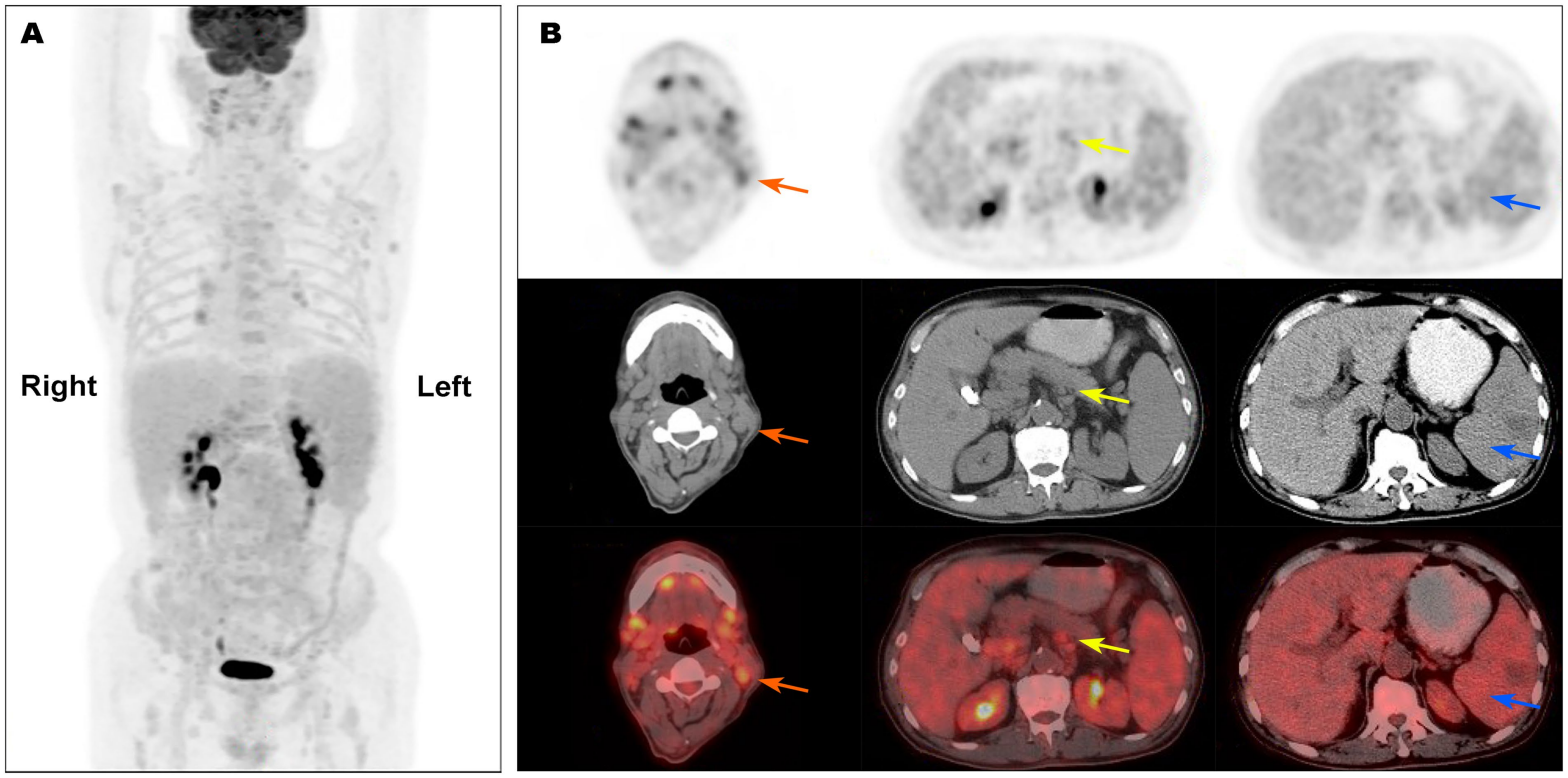
PET/CT scan showed multiple enlarged lymph nodes and splenomegaly. **(A)** showed enlarged lymph nodes above and below the diaphragm; **(B)** showed enlarged lymph nodes in cervical part (orange arrows) and abdominal part (yellow arrows), and splenomegaly (blue arrows).

On admission, physical examination showed extensive skin lesions on his chest, back, abdomen, and right inner thigh (Figure 1) and enlarged lymph nodes in the neck, axilla, and groin. Laboratory examinations revealed pancytopenia (neutrophil count, 1.81 × 10^9^/L; hemoglobin level, 91.00 g/L; platelet count, 70.00 × 10^9^/L) and remarkably higher β2-microglobulin (6.292 μg/mL). Many BPDCN cells were detected in the peripheral blood smear (60%), bone marrow aspirate smear (73.5%) (Figure 2). Meanwhile, BPDCN cells were also distributed in cervical lymph nodes, and skin dermis tissue (Figure 3). Immunophenotyping using flow cytometry (FCM) further showed a large number of BPDCN cells (54.61%), expressed positively for CD4, CD56, CD123, TCL1, and CD303, in the bone marrow (Figure 4; Table 1). Additionally, ultrasound showed multiple lymph node enlargements, with the largest node even up to 35.8 mm × 16.7 mm in the left axilla. Positron emission tomography/computed tomography (PET/CT) further demonstrated that there were multiple lymph node enlargements above and below the diaphragm, and splenomegaly (Figure 5). Next-generation sequencing (NGS) revealed multisite mutations in bone marrow cells, such as the *TET2* mutation (Table 2). Karyotype analysis confirmed a 46,XY[20] pattern. For the aforementioned symptoms and all outcomes, this patient was diagnosed with BPDCN.

**Table 1:** Immunophenotypic characterization of BPDCN cells from the patient.

Tissue type and detection method	Positive markers	Negative markers
Bone marrow (FCM)	CD2, CD4, CD7, CD10, CD36, CD45RA, CD56, CD103, CD123, CD303, HLA-DR, TCL-1, TIA-1	CD1a, CD3, cCD3, CD5, CD8, CD11c, CD13, CD16, CD19, CD20, CD22, CD23, CD25, CD26, CD30, CD33, CD34, CD38, CD45RO, CD57, CD79b, CD81, CD94, CD99, CD117, CD161, CD200, CD304, FMC7, GranzymeB, IgM, TDT, Kappa, Lambda, Perforin, sIgD, TCRgd, MPO
Lymph nodes (IHC)	CD7, CD10, CD43, CD56, CD123, TdT, Ki-67, CD21(follicular dendritic cells)	CD1α, CD3, CD4, CD8, CD15, CD20, CD34, CD68, Granzyme B, Lysozyme, MPO, PAX-5, S100, TIA-1
Skin (IHC)	CD7, CD43, CD56, CD123, Ki-67	CD3, CD4, CD20, CD34, CD38, CD68, MPO, TdT

**Table 2:** Characteristics of the gene mutation in the patient.

Gene name	Ref. Sequence	Exon	Chromosome location	Nucleotide change	Protein change	rs	Frequency
*TET2*	NM_001127208	5	4q24	c.3578G>A	p.C1193Y	-	58.00%
*TET2*	NM_001127208	11	4q24	c.4794 T>G	p.Y1598*	-	18.20%
*TET2*	NM_001127208	9	4q24	c.4160A>G	p. N1387S	-	3.90%
*TET2*	NM_001127208	3	4q24	c.2245C>T	p.Q749*	-	1.10%
*NRAS*	NM_002524	4	1p13.2	c.436G>A	p. A146T	-	4.50%
*NRAS*	NM_002524	2	1p13.2	c.34G>A	p. G12S	rs121913250	3.50%
*NRAS*	NM_002524	2	1p13.2	c.35G>A	p. G12D	rs121913237	1.70%
*BCORL1*	NM_021946	4	Xq26.1	c.469G>A	p. A157T	rs147775035	99.80%
*PDGFRA*	NM_006206	15	4q12	c.2153G>A	p. R718Q	rs367722824	66.70%
*DIS3*	NM_014953	17	13q22.1	c.2339G>C	p. R780T	-	16.60%
*CSMD1*	NM_033225	14	8p23.2	c.2012G>A	p. R671H	rs376413480	13.70%

**Figure 6:**
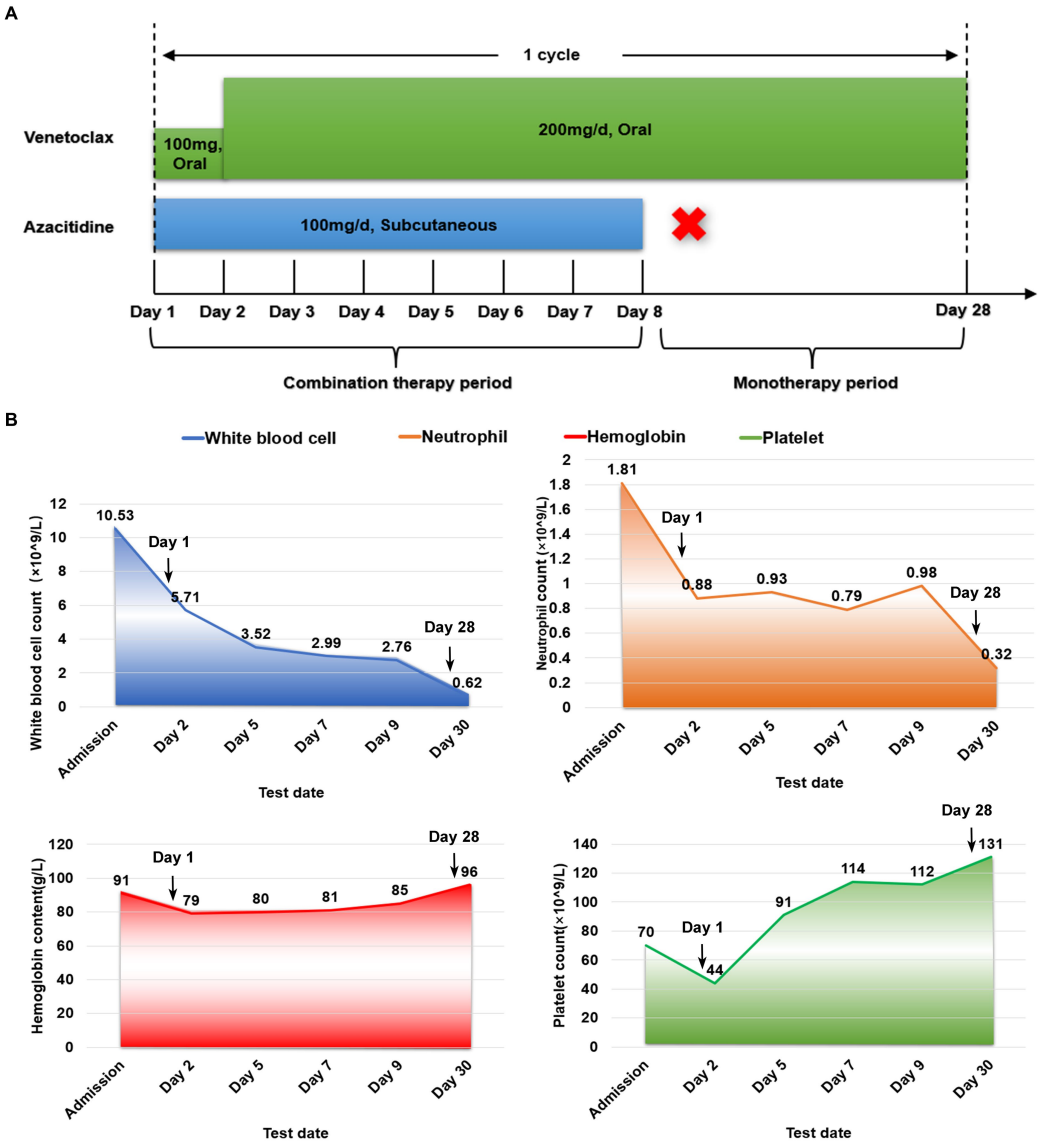
Schedules of treatment strategy and efficacy evaluation. **(A)** To avoid tumor lysis syndrome, initial dose of venetoclax was 100 mg/day oral, and the dose was increased to 200 mg/day for 27 consecutive days. Azacitidine was given with the dose of 100 mg/day for 7 consecutive days. One cycle was 4 weeks (28 days). **(B)** Efficacy evaluation for blood cell count was performed every 2 days during the combination therapy period.

Once confirmed, this patient began treatment with the oral BCL2 inhibitor venetoclax (100 mg on the first day and 200 mg for 27 consecutive days) and subcutaneously administered azacitidine (100 mg once daily for 7 consecutive days) (Figure 6A). After 9 days of treatment, the skin lesions gradually reduced in size and disappeared markedly (Figure 1). Importantly, BPDCN cells significantly decreased (4.5% in the bone marrow and 2% in the peripheral blood) after the first course of both drugs administration (Figure 2), indicating that the elderly patient presented with CR in the bone marrow. Meanwhile, the hemoglobin and platelet counts improved significantly (Figure 6B). After the first course of treatment, the patient gave up therapy and discharged from the hospital owing to his fear of the disease itself and economic factors. At about 1 month, the patient died of a possible severe infection.

## 3 Discussion

BPDCN is an extremely rare hematological malignancy. The first case of BPDCN was reported in 1994 (16), it was classified as an independent hematological tumor by the World Health Organization (WHO) in 2016 (17). According to the 2022 WHO Classification, it was classified as a histiocytic/dendritic cell neoplasm (18). BPDCN can affect multiple organs including the skin, bone marrow, lymph nodes, liver, spleen, and central nervous system (3, 6, 19). Among the common symptoms of extramedullary infiltration, 83% of patients show skin lesions (3), possibly due to the binding between the highly expressed CLA/HECA-452 in tumor cells and E-selectin in dermal endothelial cells and cutaneous T cells (5). The other common manifestations were anemia (65%), thrombocytopenia (78%), and lymphadenopathy with or without splenomegaly (61%). Meanwhile, the tonsil, lung, and eyes have also been reported to be invaded by these malignant cells (3, 6, 19). Hence, the variety of these symptoms and physical signs makes it difficult to diagnose BPDCN and delays its treatment. In addition to the manifestations, a definitive diagnosis is made based on the immunophenotype of the tumor cells, according to the 2022 WHO Classification. To begin to formally address the state of the field, the North American BPDCN Consortium was formed and defines the current standard of care and identify the most important research questions and future directions in BPDCN (10).

In the present study, when skin lesions first appeared on his chest, abdomen, and back, the patient was misdiagnosed with skin disease at a local county hospital. Although the symptoms were temporarily relieved after treatment for skin diseases, the skin lesions had recurred twice in the past year. The patient presented with extensive skin lesions, extreme fatigue, and bilateral neck and inguinal lymph node enlargement. On admission, laboratory examinations revealed pancytopenia. A peripheral blood smear, bone marrow aspirate smear, cervical lymph nodes, and skin dermal tissue showed the presence of multiple malignant cells. FCM data showed large numbers of BPDCN cells, and PET/CT further demonstrated that there were multiple lymph node enlargements above and below the diaphragm and splenomegaly. Therefore, the patient was diagnosed with BPDCN.

Once confirmed, the patient was immediately administered venetoclax + azacitidine. Previously, most patients with BPDCN received regimens such as hyper-CVAD or FLAG; canonical regimens for AML, acute lymphoblastic leukemia, or lymphoma; and targeted CD123 therapy (tagraxofusp). Although patients show good outcomes in the early stages after treatment, drug resistance and recurrence will soon occur. In addition, these regimens have many adverse reactions, especially tagraxofusp, which can lead to liver injury, thrombocytopenia, and severe capillary leakage syndrome (6–9). The most recent literature reported that patients with BPDCN treated with venetoclax (BCL2 inhibitor) and azacitidine (HMA) had confirmed clinical efficacy and safety. Importantly, in older patients with BPDCN who are unfit for chemotherapy, a combination of venetoclax + HMA may be a feasible alternative to CD123-targeted or cytotoxic chemotherapy.

*BCL2*, a key apoptotic gene, is widely expressed in various tumor cells and is closely associated with the carcinogenesis of many malignant diseases. Recently, it was reported that the BCL2 inhibitor venetoclax, a BCL2 homology 3 (BH3) mimetic, can induce apoptosis by lowering the apoptosis threshold of tumor cells (11). In 2017, Montero et al. demonstrated that BCL2 is highly expressed in BPDCN cells and that venetoclax reduced BPDCN patient-derived xenografts in mice. In clinical trials, after venetoclax treatment, enlarged lymph nodes significantly decreased, and the number of bone marrow tumor cells decreased from 85 to 44% (15). In 2019, Beziat et al. also reported that an elderly male patient with BPDCN, who still had repeated and severe skin lesions when treated with eight courses of the miniCHOP regimen, showed a tremendous therapeutic effect without tumor lysis syndrome or other adverse reactions when exchanged for venetoclax treatment (20). This suggests that venetoclax is safe, effective, and applicable in patients with BPDCN, especially in elderly patients with skin involvement.

In 2020, Piccini et al. reported a relapsed/refractory (R/R) BPDCN case with disseminated skin relapse that achieved a remarkably quick and durable response to venetoclax and HMA azacitidine treatment (21). Venetoclax alone or in combination could be used as a reference treatment for patients with BPDCN. Azacitidine, as a conventional drug in Myelodysplastic syndrome and AML, can inhibit DNA hypermethylation. In particular, 21% of the BPDCN patients were demonstrated to have a mutation in *TET2*, a methylation-related gene. In a CAL-1 cell line xenograft mouse model, azacitidine was shown to be effective in controlling BPDCN progression *in vivo* (22). Meanwhile, data from Laribi et al. and Khwaja et al. showed that two of five patients with BPDCN showed favorable effects for alleviating skin lesions after azacitidine monotherapy (23, 24). In addition, azacitidine can overcome tagraxofusp resistance by reversing the high methylation state of diphthamide biosynthesis 1 promoter region in tagraxofusp-resistant AML/BPDCN cells (25).

Although early stages of venetoclax or azacitidine alone had a good effect, most patients with BPDCN tended to develop rapid progression, and few patients achieved CR or had a chance of long-term survival. However, after treatment with a combination of venetoclax and azacitidine, all 10 patients with BPDCN successfully achieved CR or PR, and more than half had received or would receive HSCT (26). Accordingly, this combination seems to be more effective than monotherapy and makes it possible for patients to avoid transfusion dependence (21). Especially several months ago, NABC formed a consensus treatment approach for the first time that older/unfit patients who are not candidates for chemotherapy should be considered for clinical trials featuring CD123-targeted therapies alone or in combinations including HMA and/or venetoclax, or off-protocol “standard” approaches of tagraxofusp monotherapy or HMA plus venetoclax (10). In this study, the man received venetoclax (100 mg on the first day and 200 mg for 27 consecutive days) and the subcutaneous administration of azacitidine. Clinical symptoms, such as skin lesions and enlarged lymph nodes, were rapidly alleviated. In particular, the number of tumor cells in the bone marrow significantly decreased, and bone marrow CR was achieved after the first course of both drugs administration. This patient did not experience serious adverse effects such as tumor lysis syndrome or capillary leakage syndrome.

Furthermore, we comprehensively reviewed the previous literatures until January 2023, including 16 patients with BPDCN treated with venetoclax and HMA (Table 3). Among these cases (Table 4), 10 were male and 6 were female, with 87.5% of patients aged ≥60 years (age range, 22–87 years). There were 7 cases of newly diagnosed patients and 9 cases of R/R patients. Statistically, the overall response rate was 93.75% (15 of 16 patients). After the first course of treatment, the skin rash was significantly reduced in seven patients (43.75%). Meanwhile, 81.25% of patients reached CR or PR after one to five courses. Of note, in 8 cases treated with azacitidine and venetoclax combination, 6 cases (75%) showed good response to the treatment during or after the first cycle. Twelve patients experienced adverse events, including hypocytosis (six cases), renal injury (one case), gastrointestinal bleeding (two cases), and aspiration pneumonia (one case). After combination treatment, none of the patients showed severe side effects such as tumor lysis syndrome, whereas seven patients (43.75%) died due to progressive disease or uncontrollable severe infections. In this study, the elderly patient presented with CR in the bone marrow, markedly alleviated skin lesions, and reduced enlarged lymph nodes without serious adverse effects after the first course of treatment with both drugs. Based on this study and the previous data analysis, venetoclax combined with azacitidine is effective, and applicable in the treatment for BPDCN patients. Importantly, venetoclax is a small molecule that penetrates blood brain barrier, which is important for patients with BPDCN who have an increased risk of CNS involvement. But besides the treatment, side effects also need to be focused on. For example, the adverse effects of venetolax include neutropenia, gastrointestinal disorder and infections, some of which even cause life-threatening for patients. Hence, during the treatment, laboratory monitoring and supportive care should be thoughtfully scheduled.

**Table 3:** Summary of BPDCN cases treated with venetoclax and HMA.

N	Gender, Age (years)	Main lesion region	Cytogenetic/mutation status	Previous treatment	Clinical response of previous treatment	Present therapy	Clinical response of present therapy	Total course	Adverse event	HSCT	Outcome	Time from diagnosis (months)
1 (27)	Female, 65	Skin		Hyper-CVAD therapy with 20% dose reduction plus intrathecal methotrexate		DAC + VEN	Non-cardiagenic pulmonary oedema during cycle 1.	<1		No	Death	4
2 (28)	Female, 87	PB, spleen, lymph nodes, bone marrow	46, XX [22]			AZA + VEN	Complete metabolic remission showed by PET and complete hematologic remission showed by bone marrow biopsy after cycle 3.	12	Hypocytosis	No	Death	18
3 (29)	Female, 75	Skin, PB, spleen, lymph nodes, bone marrow	46, XX	Steroids	Infections	AZA + VEN	Morphogical remission confirmed by BME after 31 days and response on skin lesions in week 4.	5	Hypocytosis	No	Relapse	
4 (30)	Male, 79	Skin, bone marrow (only ringed sideroblasts)	46, XY /*SF3B1, TET2, ZRSR2*	Tagraxofusp	The course was complicated by multiple adverse events and relapsed after 6 cycles.	AZA + VEN	Major cutaneous response after 2 cycles	≥5		No	Continue to improve	
5 (21)	Male, 64	Skin, lymph nodes, bone marrow	46, XY	Hyper-CVAD	CR on skin, lymph nodes, and bone marrow. MRD was 0.01% on bone marrow detected by flow cytometry after 1 cycle, but relapsed after 3 cycles or more.	AZA + VEN	Major cutaneous response after 1 cycle, CR after 11 cycles or less	≥11	Hypocytosis	No	Persistent CR	
6 (26)	Male, 67	Skin	*ASXL1, TET2, ZRSR2*			AZA + VEN	Major cutaneous response after 1 cycle, CR after 2 cycles.	≥2	None	Yes	Remain disease-free	17
7 (26)	Female, 66	Skin	*ASXL1, VUS, SH2B3, TERT, TET2*			AZA + VEN	Major response on skin after 1 cycle, CR after 2 cycles, relapse after 5 cycles.	≥6	Hypocytosis	No	Relapse	10
8 (26)	Male, 65	Skin, lymph nodes, bone marrow	47, XY, +13 [9]/*RUNX1. SF3B1, SRSF2*			AZA + VEN	Cytogenetic and molecular remission on bone marrow, near-complete resolution of adenopathy, and major skin response after 1 cycle, relapsed on cycle 2.	<2		No	Death	2
9 (26)	Male, 78	Skin, PB, bone marrow	44, XY, add(1)(q21), der(4)t(1;4)(q21;q21), −9,-13 [9]/46,XY [11]/*ASXL1, SRSF2, TET2*	Tagraxofusp	CR on bone marrow and skin lesions response after 2 cycles, then relapsed after cycle 5.	DAC + VEN	Major response on skin after 1 cycle, CR after 4 cycles or more, then relapsed.		Hypocytosis	No	Death	10
10 (26)	Female, 22	PB, lymph nodes, bone marrow		Hyper-CVAD, cladribine, cytosine arabinoside, G-CSF, mitoxantrone, etoposide	CR, then relapsed.	DAC + VEN	Remission after 5 cycles.	6		Yes	Death	36
11 (26)	Female, 77	Skin, bone marrow	*TET2, JAK2, PHF6, SH2B3*			DAC + VEN	CR		Renal insufficiency	No	Alive	>24
12 (26)	Male, 82	Skin, lymph nodes, bone marrow, CNS	*TET2, ETV6, NRAS, TP53, U2AF1, ZRSR2*			DAC + VEN	PR on bone marrow, CNS become negative.	2		No		
13 (26)	Male, 57	Skin, bone marrow	*TET2, ASXL1*	Hyper-CVAD, alloSCT, FLAG		DAC + VEN	PR on bone marrow.	1	None	No	Alive	
14 (26)	Male, 73	Skin, lymph nodes, bone marrow	*TET2, MPL*	Hyper-CVAD, autoSCT, Tagraxofusp		DAC + VEN	CR	4	Gastrointestinal bleed	No	Alive	
15 (26)	Male, 76	Skin, bone marrow, CNS	*TET2, ASXL1, KRAS, NRAS*	Tagraxofusp		DAC + VEN	CR	3	Aspiration pneumonia, gastrointestinal bleed	No	Death	
16^#^	Male, 71	Skin, spleen, lymph nodes, bone marrow	46, XY [20]/*ASXL1, NRAS, BCORL1, PDGFRA, DIS3, CSMD1*			AZA + VEN	Major response on skin after 9 days, CR on bone marrow after 1 cycle.	1	Hypocytosis	No	Death	2

*N*, number; HSCT, hematopoietic stem cell transplantation; PB, peripheral blood; CNS, central nervous system; VUS, variants of unknown significance; alloSCT, allogeneic hematopoietic stem cell transplantation; autoSCT, autologous stem cell transplant; VEN, venetoclax; AZA, azacitidine; DAC, decitabine; BME, bone marrow examination; MRD, minimal residual disease; CR, complete response/remission; PR, partial response. ^#^The case reported in this study.

**Table 4:** Patient demographics and treatment outcomes.

Characteristic	All patients (*n* = 16)
Age, years	
<60	12.5% (2/16)
≥60	87.5% (14/16)
Sex	
Female	37.5% (6/16)
Male	62.5% (10/16)
Disease status	
Newly diagnosed	43.75% (7/16)
Relapsed or refractory	56.25% (9/16)
Main lesion region	
Skin	87.5% (14/16)
Peripheral blood	25% (4/16)
Bone marrow	81.25% (13/16)
Lymph nodes	50% (8/16)
Spleen	18.75% (3/16)
Central nervous system	12.5% (2/16)
Cytogenetic status	
Normal	31.25% (5/16)
Unnormal	12.5% (2/16)
Not report	56.25% (9/16)
Mutation status	
*TET2*	62.5% (10/16)
*ASXL1*	37.25% (6/16)
*ZRSR2*	18.75% (3/16)
Previous treatment	
Hyper-CVAD	55.56% (5/9)
Tagraxofusp	44.44% (4/9)
Other	11.11% (1/9)
Present therapy	
AZA + VEN	50% (8/16)
DAC + VEN	50% (8/16)
HSCT	
Yes	12.5% (2/16)
No	87.5% (14/16)
Outcome	
Persistent CR	6.25% (1/16)
Remain disease-free	6.25% (1/16)
Relapse	12.5% (2/16)
Death	43.75% (7/16)
Unclear/Not report	31.25% (5/16)

## 4 Conclusion

In conclusion, we reported a patient with BPDCN and disseminated skin lesions who achieved a remarkably quick response to venetoclax and azacitidine treatment. Our study and current cumulative data suggest that venetoclax combined with azacitidine is safe, effective, and applicable in the treatment of elderly patients with R/R BPDCN. Importantly, closer attention should be paid to the physical condition of patients, and the best supportive care should be given in the event of severe myelosuppression. Although NABC provides a consensus, the final therapy decision is still dependent on multiple factors, such as the drugs toxicity profile, physician preference of the patient, and drug availability and affordability in the future clinical work. This study, therefore, significantly contributes to the literature on the current and future treatment for BPDCN. Formal cost analysis of different regimens used in BPDCN remains an important goal for the consortium in the world.

## Data availability statement

The raw data supporting the conclusions of this article will be made available by the authors, without undue reservation.

## Ethics statement

Written informed consent was obtained from the individual(s) for the publication of any potentially identifiable images or data included in this article.

## Author contributions

XW: Data curation, Writing – original draft, Writing – review & editing. JG: Data curation, Writing – original draft, Writing – review & editing. YL: Data curation, Writing – original draft. NZ: Data curation, Writing – original draft. SX: Software, Writing – original draft. LW: Writing – review & editing. RY: Software, Writing – review & editing. LX: Writing – review & editing. JL: Writing – review & editing.
