# Supplementary material for: Venetoclax combined with azacitidine in blastic plasmacytoid dendritic cell neoplasm: a case report and comprehensive review on the current and future treatment
Source: Front Med (Lausanne). 2024 Jul 17;11:1425833. doi: 10.3389/fmed.2024.1425833 (PMC11288880; doi:10.3389/fmed.2024.1425833)
Supplement: Supplementary file 1 [file Data_Sheet_1.PDF]

CARE case report guidelines

CARE Checklist of information to include when writing a case report

(cc) BY-NC-ND

Topic	Item	Checklist item description	Reported on Line
**Title**	1	The diagnosis or intervention of primary focus followed by the words "case report"	Line 1
**Key Words**	2	2 to 5 key words that identify diagnoses or interventions in this case report, including "case report"	Line 38
**Abstract (no references)**	3a	Introduction: What is unique about this case and what does it add to the scientific literature?	Line 43
	3b	Main symptoms and/or important clinical findings	Line 48
	3c	The main diagnoses, therapeutic interventions, and outcomes	Line 56
	3d	Conclusion—What is the main "take-away" lesson(s) from this case?	Line 59
**Introduction**	4	One or two paragraphs summarizing why this case is unique (**may include references**)	Line 65
**Patient Information**	5a	De-identified patient specific information.	Line 99
	5b	Primary concerns and symptoms of the patient.	Line 100
	5c	Medical, family, and psycho-social history including relevant genetic information	Line 109
	5d	Relevant past interventions with outcomes	Line 101
**Clinical Findings**	6	Describe significant physical examination (PE) and important clinical findings.	Line 130
**Timeline**	7	Historical and current information from this episode of care organized as a timeline	Line 101
**Diagnostic Assessment**	8a	Diagnostic testing (such as PE, laboratory testing, imaging, surveys).	Line 111
	8b	Diagnostic challenges (such as access to testing, financial, or cultural)	Not applicable
	8c	Diagnosis (including other diagnoses considered)	Line 126
	8d	Prognosis (such as staging in oncology) where applicable	Not applicable
**Therapeutic Intervention**	9a	Types of therapeutic intervention (such as pharmacologic, surgical, preventive, self-care)	Line 128
	9b	Administration of therapeutic intervention (such as dosage, strength, duration)	Line 128
	9c	Changes in therapeutic intervention (with rationale)	Not applicable
**Follow-up and Outcomes**	10a	Clinician and patient-assessed outcomes (if available)	Line 130
	10b	Important follow-up diagnostic and other test results	Not applicable
	10c	Intervention adherence and tolerability (How was this assessed?)	Line 135
	10d	Adverse and unanticipated events	Line 221
**Discussion**	11a	A scientific discussion of the strengths AND limitations associated with this case report	Line 141
	11b	Discussion of the relevant medical literature **with references**.	Line 141
	11c	The scientific rationale for any conclusions (including assessment of possible causes)	Line 224
	11d	The primary "take-away" lessons of this case report (without references) in a one paragraph conclusion	Line 239
**Patient Perspective**	12	The patient should share their perspective in one to two paragraphs on the treatment(s) they received	Not applicable
**Informed Consent**	13	Did the patient give informed consent? Please provide if requested	Yes ☑ No ☐
